# ATA 2015-2025 risk stratification transition: prognostic significance of N1b disease in papillary thyroid carcinoma with limited nodal burden

**DOI:** 10.1093/oncolo/oyag186

**Published:** 2026-05-12

**Authors:** Ziqiang Wang, Yangyang Xie, Tingting Wang, Danwei Du, Xue Song

**Affiliations:** Department of Thyroid and Breast Surgery, The First People’s Hospital of Xiaoshan District, Xiaoshan Affiliated Hospital of Wenzhou Medical University, Hangzhou, 311000, China; Zhejiang Key Laboratory of Multi-omics Precision Diagnosis and Treatment of Liver Diseases, Department of General Surgery, Sir Run-Run Shaw Hospital, Zhejiang University School of Medicine, Hangzhou, 310016, China; The Department of General Practice, The Second Affiliated Hospital and Yuying Children’s Hospital of Wenzhou Medical University, Wenzhou, 325027, China; Department of Anorectal, Hangzhou TCM Hospital Affiliated to Zhejiang Chinese Medical University, Hangzhou, 310000, China; Department of Respiratory and Critical Care Medicine, Hangzhou TCM Hospital Affiliated to Zhejiang Chinese Medical University, Hangzhou, 310007, China

**Keywords:** papillary thyroid carcinoma, N1b disease, low-volume nodal metastases, ATA risk stratification, competing-risk analysis, SEER

## Abstract

**Background:**

The ATA risk stratification system has evolved from the 2015 framework toward a more composite nodal risk assessment in the 2025 update. Whether low-volume lateral neck disease remains clinically meaningful is uncertain. We evaluated the prognostic significance of N1b vs N1a disease in papillary thyroid carcinoma (PTC) with ≤5 metastatic lymph nodes.

**Materials and Methods:**

We identified 11 878 patients with PTC and N1a or N1b disease with ≤5 metastatic lymph nodes from the SEER database (2000-2022). One-to-one propensity score matching was performed. OS and CSS were assessed using Kaplan–Meier analysis, and CSD and OCD using cumulative incidence functions and Fine–Gray models.

**Results:**

Among eligible patients, 9392 had N1a disease and 2486 had N1b disease. After matching, 2467 patients remained in each group. N1b was associated with significantly worse OS, CSS, and CSD in both unmatched and matched cohorts. In the matched cohort, 5-year CSD increased from 1.5% to 2.8%. On multivariable Fine–Gray analysis, N1b remained independently associated with higher CSD risk (SHR, 2.52; 95% CI, 1.85-3.43; *P* < .001). The adverse effect was more evident in patients with positive ETE, tumor diameter >2 cm, and older age.

**Conclusion:**

Among patients with PTC and ≤5 metastatic lymph nodes, N1b retains independent adverse prognostic significance despite low nodal burden. Low-volume nodal disease should not be considered uniformly low risk when lateral neck involvement is present, particularly in patients with positive ETE, tumor diameter >2 cm, or older age.

Implications for Practice In papillary thyroid carcinoma with ≤5 metastatic lymph nodes, lateral neck involvement remained associated with worse cancer-specific outcomes despite limited nodal burden. These findings support postoperative risk assessment that considers nodal location in addition to nodal count, particularly when extrathyroidal extension, larger tumor size, or older age is present. The results should be interpreted as complementary long-term outcome evidence rather than direct recurrence-based validation of risk stratification frameworks.

## Introduction

Papillary thyroid carcinoma (PTC) is the most common endocrine malignancy and is generally associated with an excellent prognosis; however, the risk of recurrence and the need for long-term management are not uniform across patients.^1^^,^^2^ For PTC, AJCC/TNM staging alone is insufficient to fully guide postoperative management.^3^ Accordingly, the initial recurrence risk stratification system proposed by the American Thyroid Association (ATA) plays a central role in clinical practice. This framework is used not only to estimate the initial risk of recurrence, but also to guide the intensity of postoperative thyroid-stimulating hormone (TSH) suppression, the frequency of imaging and biochemical surveillance, and the use of adjuvant therapies.^4^^,^^5^ As a result, changes in the boundaries of risk categories may have direct and important clinical implications.

Lymph node metastasis is a key component of ATA initial recurrence risk stratification, yet its prognostic significance has long been recognized as heterogeneous.^6^^,^^7^ The 2015 ATA guidelines explicitly incorporated both the number and size/volume of nodal metastases into risk assessment: patients with ≤5 micrometastatic lymph node foci (<0.2 cm) could be classified as low risk, whereas clinically apparent nodal disease or >5 pathologic positive lymph nodes (with the largest metastatic focus <3 cm) were generally categorized as intermediate risk.^8^^,^^9^ In contrast, the ATA 2025 framework further refines the traditional 3-tier model into 4 categories—low, low-intermediate, intermediate-high, and high risk—and places greater emphasis on the anatomic location of nodal disease, the size and overall burden of metastases, and the cumulative effect of additional adverse features. Within this updated framework, low-volume central compartment nodal metastasis may correspond to a lower-risk category, whereas clinically apparent lateral neck metastasis (cN1b) <3 cm is classified as intermediate-high risk.^5^ These changes suggest that nodal risk assessment is evolving from a relatively count-based approach toward a more refined composite evaluation, and that “low-volume” disease does not necessarily equate to “low-risk” disease, particularly when the patient also has the anatomic features of N1b involvement.^10^^,^^11^

This evolution raises a clinically relevant but incompletely answered question: among patients with N1b disease and ≤5 metastatic lymph nodes, should low nodal burden be interpreted as indicating limited risk, or does the presence of lateral neck involvement still identify a subgroup at higher risk of adverse outcomes that warrants closer attention during postoperative risk reassessment? Direct evidence addressing this question remains limited.^12^ Although previous studies have generally associated N1b disease with a higher risk of recurrence, most have focused on broad N1b populations or have used recurrence as the primary endpoint, with relatively little attention paid specifically to the setting of low nodal burden.^13^^,^^14^ At the same time, evidence directly comparing long-term survival outcomes between N1a and N1b disease among patients with ≤5 metastatic lymph nodes remains insufficient.^15^ Moreover, given the overall favorable prognosis of PTC, non-cancer death may act as a competing event in conventional survival analyses. Therefore, evaluating the true prognostic significance of nodal stage from the perspective of long-term adverse outcomes may provide important complementary evidence for refining risk stratification and postoperative management in patients with low-volume nodal metastases.^16^

Against this background, we used the SEER database to focus on patients with PTC and ≤5 metastatic lymph nodes. Within propensity score–matched and competing-risk frameworks, we systematically compared overall survival, cancer-specific survival, and cancer-specific death between N1a and N1b disease, and further examined whether the adverse prognostic impact of N1b was concentrated in more aggressive clinical subgroups, including those with larger tumor size, extrathyroidal extension, and older age.^17^ By addressing this question, we aimed to provide complementary evidence for postoperative risk assessment and follow-up stratification in patients with low-volume nodal metastases, and to inform individualized management decisions, including the intensity of TSH suppression.^18^

## Methods

### Data source and study population

This population-based retrospective cohort study used data from the SEER database maintained by the National Cancer Institute. Patients with PTC diagnosed between January 1, 2000 and December 31, 2022 were identified using SEER*Stat. Because SEER is a publicly available de-identified database, institutional review board approval and informed consent were not required.

**Figure 1.**
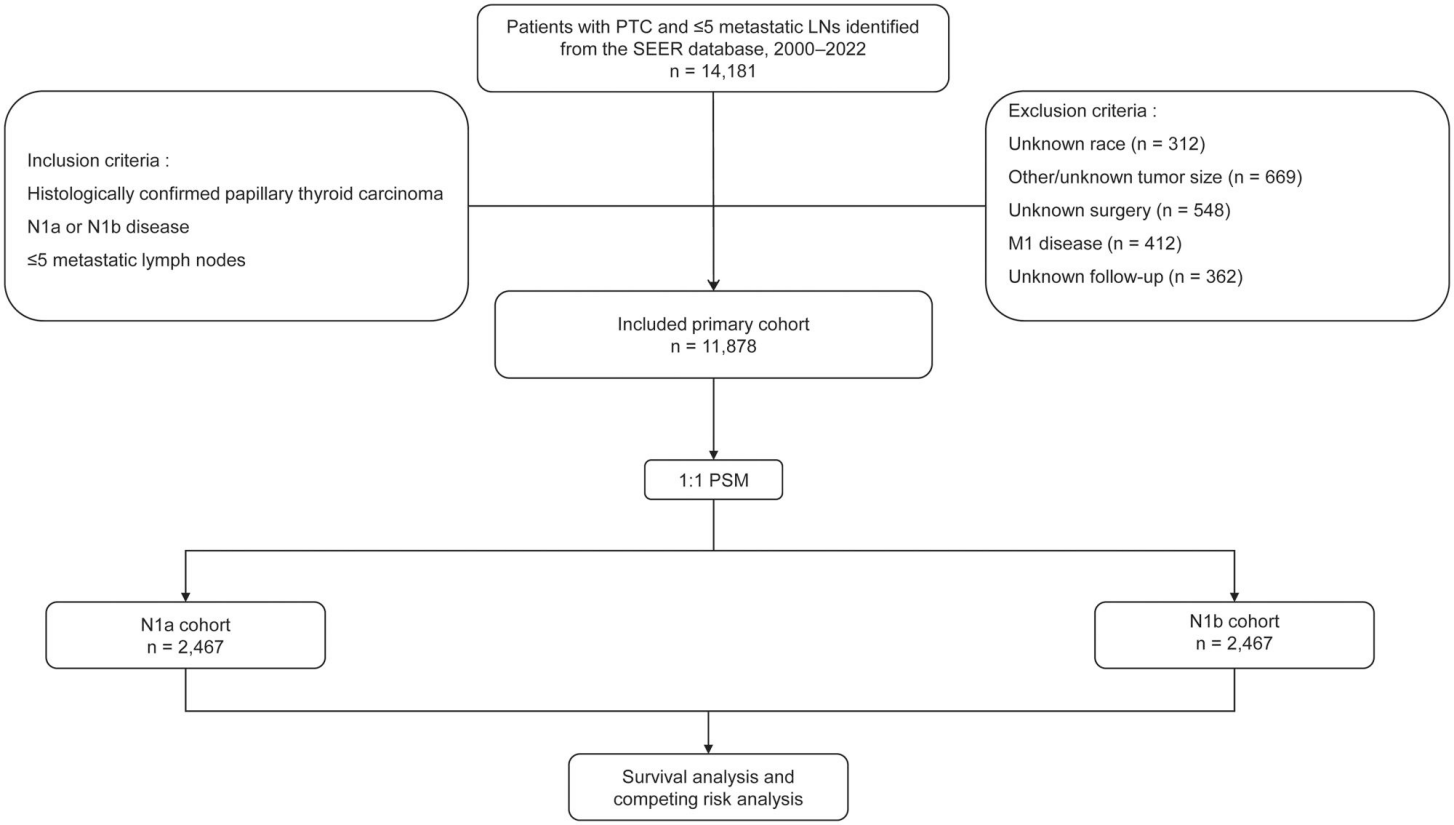
Flowchart of patient selection for the study cohort of papillary thyroid carcinoma with low-volume nodal metastases. Abbreviations: LN, lymph node; PSM, propensity score matching; PTC, papillary thyroid carcinoma; SEER, Surveillance, Epidemiology, and End Results.

The study was designed to compare prognosis between N1a and N1b disease in the setting of low nodal burden (≤5 metastatic lymph nodes). We first identified patients with PTC and regional lymph node metastasis recorded as N1a or N1b in SEER, and then restricted the cohort to those with ≤5 metastatic lymph nodes. A total of 14 181 cases met the initial screening criteria. Inclusion criteria were (1) histologically confirmed PTC; (2) nodal stage recorded as N1a or N1b; (3) ≤5 metastatic lymph nodes; (4) no distant metastasis; and (5) available survival data. Patients were excluded for unknown race (*n* = 312), tumor size coded as other/unknown (*n* = 669), unknown surgical procedure (*n* = 548), distant metastasis (M1, *n* = 412), or unavailable follow-up information (*n* = 362). Ultimately, 11 878 patients were included (Figure 1).

### Variable definitions

The primary exposure was nodal stage (N1a vs N1b). N1a was defined as metastasis to central compartment lymph nodes, whereas N1b was defined as metastasis to lateral cervical and/or superior mediastinal lymph nodes. To reduce ambiguity arising from possible inconsistencies between clinical and pathologic staging in SEER, the recorded N1a/N1b classification was used uniformly, without distinguishing cN1b from pN1b.

Baseline covariates included demographic, tumor-related, treatment-related, and socioeconomic variables. Demographic variables were age (<55 vs ≥55 years), sex, race (White vs non-White), and marital status (married vs unmarried). Marital status was included as a sociodemographic covariate reflecting potential differences in social support and access to care, rather than as a thyroid cancer–specific clinical factor. Tumor-related variables included ETE, tumor size (≤1 cm, >1–2 cm, >2–4 cm, and >4 cm), number of metastatic lymph nodes (1–2 vs 3–5), and multifocality (single vs multi). Treatment-related variables included surgical procedure [lobectomy (LT) vs total thyroidectomy (TT)] and radioactive iodine (RAI) therapy. Socioeconomic variables included household income level (low, middle, high) and residential area (urban vs rural).

Because ETE and multifocality had a relatively high proportion of missing values, complete-case analysis would have markedly reduced the sample size and potentially introduced selection bias. Therefore, unknown values were retained as separate categories in the primary analysis and incorporated into propensity score matching (PSM) and multivariable models.

OS was defined as time from diagnosis to death from any cause, with surviving patients censored at last follow-up. CSS was defined as time from diagnosis to thyroid cancer–related death, with non-cancer deaths treated as censored events. In the competing-risk framework, CSD was defined as death attributed to thyroid cancer, whereas other-cause death (OCD) was defined as death from any other cause. Given the favorable prognosis of PTC and the non-negligible proportion of non-cancer deaths, CSD was prespecified as the primary endpoint, whereas CSS was used mainly for comparison with conventional survival analyses.

### Propensity score matching

Because assignment to the N1a and N1b groups was not random, PSM was performed to reduce confounding. Propensity scores were estimated using multivariable logistic regression including all prespecified baseline covariates, with the probability of N1b classification defined as the propensity score. A 1:1 nearest-neighbor matching approach without replacement was applied. Covariate balance before and after matching was assessed using standardized mean differences (SMDs), with absolute SMD <0.10 indicating adequate balance. Changes in balance were visualized using a Love plot (Figure S1, see online supplementary material for a color version of this figure).

### Survival and competing-risk analyses

In both the unmatched and matched cohorts, Kaplan–Meier methods were used to estimate OS and CSS, and differences between N1a and N1b groups were compared using the log-rank test.

Within the competing-risk framework, cumulative incidence functions (CIFs) were used to estimate CSD and OCD, and Gray’s test was used for between-group comparisons. The 1-, 3-, and 5-year cumulative incidence rates of CSD and OCD were reported in both cohorts.

To assess independent predictors of CSD, a multivariable Fine–Gray subdistribution hazards model was constructed in the matched cohort. Variables entered into the model included age, sex, race, marital status, ETE, tumor size, number of metastatic lymph nodes, multifocality, RAI therapy, household income, residential area, and nodal stage. Subdistribution hazard ratios (SHRs) with 95% CIs were reported.

### Subgroup analyses

Prespecified subgroup analyses were conducted in the matched cohort to evaluate whether the prognostic difference between N1a and N1b varied across clinical settings. Main-text subgroups included ETE (no vs yes), tumor size (≤1 cm, >1-2 cm, >2-4 cm, and >4 cm), and age (<55 vs ≥55 years). Competing-risk analyses stratified by metastatic lymph node number (1-2 vs 3-5) were presented as supplementary analyses. Additional subgroup analyses according to sex, race, marital status, multifocality, RAI status, household income, and residential area were also performed, but were not shown individually in the main text.

### Statistical analysis

All analyses were performed using R software. PSM was conducted using the MatchIt package; balance assessment and Love plots were generated using cobalt; Kaplan–Meier analyses were performed using survival and survminer; and competing-risk analyses, including CIF estimation, Gray’s test, and Fine–Gray regression, were conducted using cmprsk. All tests were 2-sided, and *P* < .05 was considered statistically significant.

## Results

### Baseline characteristics and propensity score matching

A total of 11 878 patients with PTC and ≤5 metastatic lymph nodes were included, including 9392 (79.1%) with N1a disease and 2486 (20.9%) with N1b disease. In the unmatched cohort, most patients were aged <55 years (67.4%), female (71.9%), and White (80.8%). Tumor size was most commonly >1-2 cm (38.0%) or >2-4 cm (28.7%), and 63.9% of patients had 1-2 metastatic lymph nodes, whereas 36.1% had 3-5 (Table 1).

**Table 1.** Baseline clinicopathological characteristics of patients with PTC and ≤5 metastatic LNs stratified by N stage (N1a vs N1b) before and after PSM.

Characteristics	Before PSM			*P* value	After PSM			*P* value
	**All**	**N1a**	**N1b**		**All**	**N1a**	**N1b**	
	***N* = 11 878**	***N* = 9392**	***N* = 2486**		***N* = 4934**	***N* = 2467**	***N* = 2467**	
**Age**				<.001				.415
** <55**	8003 (67.4%)	6521 (69.4%)	1482 (59.6%)		2987 (60.5%)	1508 (61.1%)	1479 (60.0%)	
** ≥55**	3875 (32.6%)	2871 (30.6%)	1004 (40.4%)		1947 (39.5%)	959 (38.9%)	988 (40.0%)	
**Gender**				<.001				.881
** Female**	8541 (71.9%)	6915 (73.6%)	1626 (65.4%)		3254 (66.0%)	1630 (66.1%)	1624 (65.8%)	
** Male**	3337 (28.1%)	2477 (26.4%)	860 (34.6%)		1680 (34.0%)	837 (33.9%)	843 (34.2%)	
**Race**				.660				.884
** White**	9598 (80.8%)	7581 (80.7%)	2017 (81.1%)		4005 (81.2%)	2005 (81.3%)	2000 (81.1%)	
** Non-White**	2280 (19.2%)	1811 (19.3%)	469 (18.9%)		929 (18.8%)	462 (18.7%)	467 (18.9%)	
**Marital status**				.739				.930
** Married**	7106 (59.8%)	5611 (59.7%)	1495 (60.1%)		2966 (60.1%)	1485 (60.2%)	1481 (60.0%)	
** Unmarried**	4772 (40.2%)	3781 (40.3%)	991 (39.9%)		1968 (39.9%)	982 (39.8%)	986 (40.0%)	
**ETE**				<.001				.847
** No**	4046 (34.1%)	3221 (34.3%)	825 (33.2%)		1655 (33.5%)	836 (33.9%)	819 (33.2%)	
** Unknown**	5662 (47.7%)	4577 (48.7%)	1085 (43.6%)		2150 (43.6%)	1073 (43.5%)	1077 (43.7%)	
** Yes**	2170 (18.3%)	1594 (17.0%)	576 (23.2%)		1129 (22.9%)	558 (22.6%)	571 (23.1%)	
**Surgery**				<.001				.087
** LT**	602 (5.1%)	534 (5.7%)	68 (2.7%)		113 (2.3%)	47 (1.9%)	66 (2.7%)	
** TT**	11 276 (94.9%)	8858 (94.3%)	2418 (97.3%)		4821 (97.7%)	2420 (98.1%)	2401 (97.3%)	
**Tumor size, cm**				<.001				.743
** ≤1**	2789 (23.5%)	2033 (21.6%)	756 (30.4%)		1471 (29.8%)	730 (29.6%)	741 (30.0%)	
** 1-2**	4518 (38.0%)	3701 (39.4%)	817 (32.9%)		1667 (33.8%)	850 (34.5%)	817 (33.1%)	
** 2-4**	3407 (28.7%)	2803 (29.8%)	604 (24.3%)		1199 (24.3%)	597 (24.2%)	602 (24.4%)	
** >4**	1164 (9.8%)	855 (9.1%)	309 (12.4%)		597 (12.1%)	290 (11.8%)	307 (12.4%)	
**LN positive**				<.001				.753
** 1-2**	7587 (63.9%)	6446 (68.6%)	1141 (45.9%)		2266 (45.9%)	1127 (45.7%)	1139 (46.2%)	
** 3-5**	4291 (36.1%)	2946 (31.4%)	1345 (54.1%)		2668 (54.1%)	1340 (54.3%)	1328 (53.8%)	
**Multifocal**				<.001				.934
** Multi**	3265 (27.5%)	2484 (26.4%)	781 (31.4%)		1539 (31.2%)	765 (31.0%)	774 (31.4%)	
** Single**	2865 (24.1%)	2272 (24.2%)	593 (23.9%)		1190 (24.1%)	600 (24.3%)	590 (23.9%)	
** Unknown**	5748 (48.4%)	4636 (49.4%)	1112 (44.7%)		2205 (44.7%)	1102 (44.7%)	1103 (44.7%)	
**Radiotherapy**				<.001				.454
** No**	4156 (35.0%)	3410 (36.3%)	746 (30.0%)		1457 (29.5%)	716 (29.0%)	741 (30.0%)	
** Yes**	7722 (65.0%)	5982 (63.7%)	1740 (70.0%)		3477 (70.5%)	1751 (71.0%)	1726 (70.0%)	
**Household income**				.763				.964
** High**	3213 (27.1%)	2555 (27.2%)	658 (26.5%)		1316 (26.7%)	658 (26.7%)	658 (26.7%)	
** Low**	319 (2.7%)	252 (2.7%)	67 (2.7%)		127 (2.6%)	62 (2.5%)	65 (2.6%)	
** Middle**	8346 (70.3%)	6585 (70.1%)	1761 (70.8%)		3491 (70.8%)	1747 (70.8%)	1744 (70.7%)	
**County type**				.016				.564
** Rural**	868 (7.3%)	658 (7.0%)	210 (8.4%)		396 (8.0%)	192 (7.8%)	204 (8.3%)	
** Urban**	11 010 (92.7%)	8734 (93.0%)	2276 (91.6%)		4538 (92.0%)	2275 (92.2%)	2263 (91.7%)	

Abbreviations: ETE, extrathyroidal extension; LN, lymph node; PSM, propensity score matching; RAI, radioactive iodine.

Before matching, significant baseline differences existed between groups. Compared with N1a disease, N1b disease was more often associated with age ≥55 years (40.4% vs 30.6%, *P* < .001), male sex (34.6% vs 26.4%, *P* < .001), positive ETE (23.2% vs 17.0%, *P* < .001), tumor size >4 cm (12.4% vs 9.1%, *P* < .001), and 3-5 metastatic lymph nodes (54.1% vs 31.4%, *P* < .001). Patients with N1b disease were also more likely to undergo TT (97.3% vs 94.3%, *P* < .001) and receive RAI (70.0% vs 63.7%, *P* < .001).

After 1:1 PSM, 4934 patients remained in the matched cohort, with 2467 in each group. All baseline variables were well balanced after matching (all *P* > .05), and all included covariates had SMDs < 0.1 (Table 1; Figure S1, see online supplementary material for a color version of this figure). Median follow-up for OS analysis was 76 months in the overall cohort and 88 months in the matched cohort. For competing-risk analyses, the corresponding median follow-up times were 58 and 85 months.

### Overall survival and cancer-specific survival

**Figure 2.**
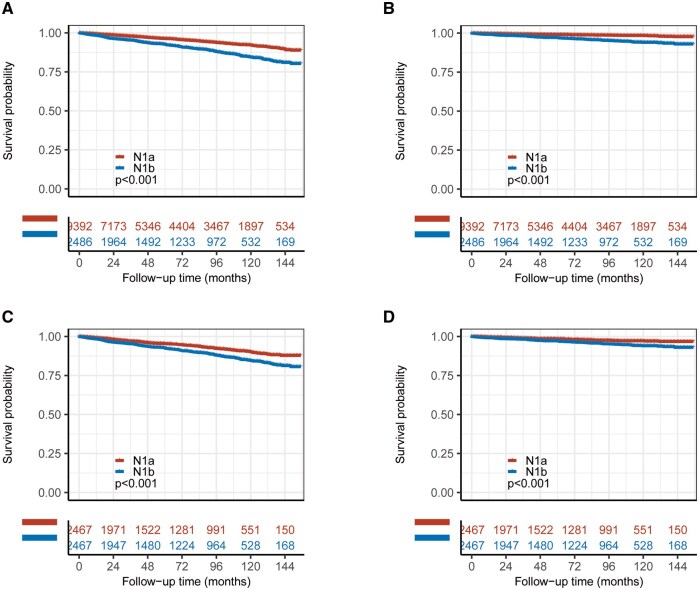
Overall survival and cancer-specific survival according to nodal stage in patients with PTC and ≤5 metastatic lymph nodes. (A) OS in the unmatched cohort. (B) CSS in the unmatched cohort. (C) OS in the PSM cohort. (D) CSS in the PSM cohort. Survival curves were estimated using the Kaplan–Meier method and compared between the N1a and N1b groups using the log-rank test. Abbreviations: CSS, cancer-specific survival; OS, overall survival; PSM, propensity score matching; PTC, papillary thyroid carcinoma.

Kaplan–Meier analyses showed that both OS and CSS were significantly worse in patients with N1b disease than in those with N1a disease in both the unmatched and matched cohorts (Figure 2). In the unmatched cohort, OS and CSS curves separated early and continued to diverge over time (both log-rank *P* < .001). After matching, the differences remained significant for both OS and CSS (both log-rank *P* < .001), indicating that the adverse prognostic effect of N1b was not fully explained by baseline imbalance.

### Competing-risk analysis of CSD and OCD

**Figure 3.**
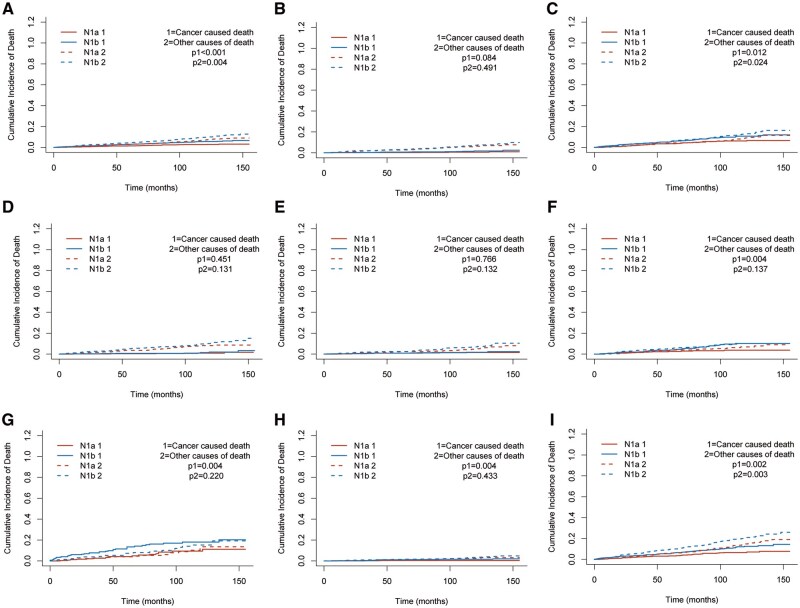
Cumulative incidence of CSD and OCD by nodal stage in the matched cohort and prespecified subgroups of patients with PTC and ≤5 metastatic lymph nodes. (A) Overall matched cohort. (B) ETE-negative subgroup. (C) ETE-positive subgroup. (D) Tumor size ≤1 cm. (E) Tumor size >1-2 cm. (F) Tumor size >2-4 cm. (G) Tumor size >4 cm. (H) Age <55 years. (I) Age ≥55 years. Cumulative incidence functions were estimated within a competing-risk framework, and differences between the N1a and N1b groups were compared using Gray’s test. Abbreviations: CSD, cancer-specific death; ETE, extrathyroidal extension; OCD, other-cause death; PTC, papillary thyroid carcinoma.

Given the favorable prognosis of PTC and the occurrence of non-cancer deaths, we further evaluated CSD and OCD using competing-risk methods (Table 2; Figure 3A). In the unmatched cohort, the 1-, 3-, and 5-year CSD rates were 0.30%, 0.60%, and 0.90% in the N1a group, compared with 0.80%, 1.80%, and 2.80% in the N1b group (Gray’s test, *P* < .001). Over the same intervals, OCD rates were 0.40%, 1.50%, and 2.80% in N1a, vs 0.80%, 2.90%, and 4.60% in N1b (*P* < .001).

**Table 2.** Cumulative incidence of CSD and OCD in patients with PTC and ≤5 metastatic LNs according to N stage (N1a vs N1b) before and after PSM.

	Cancer-specific death (%)			*P* value	Other causes death (%)			*P* value
	1-year CIF	3-year CIF	5-year CIF		1-year CIF	3-year CIF	5-year CIF	
**Before PSM**								
** N1a**	0.30	0.60	0.90	<.001	0.40	1.50	2.80	<.001
** N1b**	0.80	1.80	2.80		0.80	2.90	4.60	
**After PSM**								
** N1a**	0.40	1.00	1.50	<.001	0.50	1.90	3.00	.004
** N1b**	0.80	1.80	2.80		0.80	2.90	4.60	

Abbreviations: CSD, cancer-specific death; OCD, other cause death; PSM, propensity score matching; PTC, papillary thyroid carcinoma; SEER, Surveillance, Epidemiology, and End Results.

This pattern persisted after matching. In the matched cohort, the 1-, 3-, and 5-year CSD rates were 0.40%, 1.00%, and 1.50% in the N1a group, compared with 0.80%, 1.80%, and 2.80% in the N1b group (*P* < .001). The corresponding OCD rates were 0.50%, 1.90%, and 3.00% in N1a vs 0.80%, 2.90%, and 4.60% in N1b (*P* = .004). Thus, patients with N1b disease had higher cumulative incidences of both thyroid cancer–related and non-cancer death, but the more pronounced and consistent difference remained concentrated in CSD.

### Multivariable fine–gray model for CSD

A multivariable Fine–Gray model was constructed in the matched cohort (Table 3). Age ≥55 years was one of the strongest adverse factors for CSD (SHR, 7.15; 95% CI, 4.83-10.56; *P* < .001). Unmarried status, included as a sociodemographic covariate, was associated with a modestly higher CSD risk (SHR, 1.38; 95% CI, 1.03-1.84; *P* = .033). Positive extrathyroidal extension was also associated with increased CSD risk (SHR, 4.22; 95% CI, 2.77-6.42; *P* < .001).

**Table 3.** Multivariable Fine–Gray subdistribution hazard analysis for CSD in patients with PTC and ≤5 metastatic LNs according to N stage (N1a vs N1b).

Characteristics	Subdistribution proportion hazards model		
	SHR	95% CI	*P* value
**Age**			
** <55**	Reference		
** ≥55**	7.15	4.83-10.56	<.001
**Gender**			
** Female**	Reference		
** Male**	1.35	0.99-1.82	.055
**Race**			
** White**	Reference		
** Non-White**	0.98	0.67-1.44	.930
**Marital status**			
** Married**	Reference		
** Unmarried**	1.38	1.03-1.84	.033
**ETE**			
** No**	Reference		
** Unknown**	0.93	0.31-2.83	.900
** Yes**	4.22	2.77-6.42	<.001
**Tumor size, cm**			
** ≤1**	Reference		
** 1-2**	1.22	0.69-2.15	.490
** 2-4**	2.99	1.79-5.01	<.001
** >4**	5.34	3.13-9.09	<.001
**LN positive**			
** 1-2**	Reference		
** 3-5**	1.03	0.76-1.39	.860
**Multifocal**			
** Multi**	Reference		
** Single**	1.15	0.82-1.61	.410
** Unknown**	2.53	0.89-7.18	.081
**Radiotherapy**			
** No**	Reference		
** Yes**	0.46	0.34-0.62	<.001
**Household income**			
** High**	Reference		
** Low**	1.60	0.58-4.43	.370
** Middle**	1.61	1.07-2.43	.023
**County type**			
** Rural**	Reference		
** Urban**	1.06	0.57-1.98	.840
**Group**			
** N1a**	Reference		
** N1b**	2.52	1.85-3.43	<.001

Abbreviations: CSD, cancer-specific death; ETE, extrathyroidal extension; OCD, other-cause death; PSM, propensity score matching; PTC, papillary thyroid carcinoma; SHR, subdistribution hazard ratio.

Tumor size demonstrated a clear risk gradient. Compared with tumors ≤1 cm, tumors >2-4 cm (SHR, 2.99; 95% CI, 1.79-5.01; *P* < .001) and >4 cm (SHR, 5.34; 95% CI, 3.13-9.09; *P* < .001) were significantly associated with higher CSD, whereas tumors >1-2 cm were not (*P* = .490). Notably, within this cohort restricted to ≤5 metastatic lymph nodes, having 3-5 nodes rather than 1-2 was not an independent adverse factor (SHR, 1.03; 95% CI, 0.76-1.39; *P* = .860), suggesting that numerical differences alone were insufficient to explain prognostic heterogeneity in the low-burden setting. RAI therapy was independently associated with lower CSD risk (SHR, 0.46; 95% CI, 0.34-0.62; *P* < .001).

Importantly, N1b stage remained an independent risk factor for CSD after adjustment (SHR, 2.52; 95% CI, 1.85-3.43; *P* < .001), indicating that even among patients with low-volume nodal metastases, lateral neck involvement retained clear adverse prognostic significance.

### Prespecified subgroup analyses

Competing-risk subgroup analyses were performed in the matched cohort to explore effect heterogeneity (Figure 3B–I). Stratified by ETE, no significant between-group difference in CSD was observed in patients without ETE (*P*1 = .084), and OCD was likewise not different (*P*2 = .491; Figure 3B). In contrast, among patients with positive ETE, N1b disease was associated with significantly higher CSD (*P*1 = .012) and OCD (*P*2 = .024; Figure 3C), suggesting that the adverse effect of N1b was more prominent in locally invasive disease.

Stratified by tumor size, no significant CSD difference between N1a and N1b disease was observed in tumors ≤1 cm (*P*1 = .451) or >1-2 cm (*P*1 = .766), and OCD differences were also not significant (Figure 3D and E). However, in the >2-4 cm and >4 cm subgroups, N1b disease was associated with significantly higher CSD (both *P*1 = .004), whereas OCD remained nonsignificant (Figure 3F and G). These findings suggest that the adverse cancer-specific effect of N1b becomes more evident as tumor size increases.

When stratified by age, N1b disease was associated with higher CSD in patients aged <55 years (*P*1 = .004), without a significant difference in OCD (*P*2 = .433; Figure 3H). Among patients aged ≥55 years, N1b remained associated with higher CSD and higher OCD (*P*1 = .002, *P*2 = .003; Figure 3I). Overall, the adverse prognostic effect of N1b was not homogeneous, but was mainly concentrated in clinically more aggressive or higher-risk subgroups, particularly those with positive ETE, tumor diameter >2 cm, and older age.

Supplementary analyses further showed that N1b disease was associated with higher CSD in both the 1-2 and 3-5 metastatic lymph node subgroups (Figure S2, see online supplementary material for a color version of this figure). Across other supplementary subgroup analyses, the direction of effect was generally consistent, although statistical significance was not uniform.

## Discussion

In this large SEER-based study, we focused on patients with PTC and ≤5 metastatic lymph nodes and systematically compared long-term outcomes between N1a and N1b disease. Under both conventional survival analysis and competing-risk frameworks, N1b disease was associated with significantly worse OS, CSS, and CSD. After PSM and multivariable Fine–Gray adjustment, N1b remained independently associated with higher CSD risk (SHR, 2.52; 95% CI, 1.85-3.43; *P* < .001). In parallel, the 5-year cumulative incidence of CSD increased from 1.50% in the N1a group to 2.80% in the N1b group after matching.^19^ Together, these findings indicate that even in the setting of low nodal burden, lateral neck involvement conveys adverse prognostic information beyond nodal count alone.^20^

A key implication is that low-volume disease does not necessarily equate to low risk. In practice, patients with relatively few metastatic lymph nodes are often intuitively regarded as having limited risk. However, our results show that when such patients also have the anatomic pattern of N1b disease, their long-term risk of thyroid cancer–related death remains significantly increased.^21^ This may explain why, in our cohort, 3-5 metastatic lymph nodes did not emerge as an independent adverse factor, whereas N1b status did. Within the low-burden range, numerical differences alone may be insufficient to explain prognostic heterogeneity, whereas lateral neck involvement may carry greater stratification value.^22^

These findings are directionally aligned with the ATA emphasis on composite risk assessment. The 2015 ATA guidelines incorporated nodal number and size/volume into initial risk assessment, whereas ATA 2025 further refines the framework and places greater weight on nodal location, metastatic burden, and additional adverse features. Within this updated system, clinically apparent lateral neck metastasis (cN1b) <3 cm is classified as intermediate-high risk.^5^ Our results cannot directly validate specific ATA 2025 category boundaries because SEER lacks important variables, including metastatic node size, extranodal extension, and the burden of clinically apparent nodal disease. Nevertheless, our findings support the view that low nodal count alone should not lead to underestimation of risk when N1b disease is present.^23^ Therefore, our results are broadly consistent with the direction of the 2025 ATA framework. At the same time, they suggest that future refinements of risk stratification may further consider the heterogeneity of low-volume N1b disease by integrating nodal location with modifiers such as extrathyroidal extension, primary tumor size, patient age, extranodal extension, metastatic deposit size, postoperative thyroglobulin, and structural recurrence when such information is available.

Most previous studies associating N1b disease with worse prognosis did not specifically focus on patients with ≤5 metastatic lymph nodes. By narrowing the question to this clinically relevant gray-zone population, our study provides more direct evidence for risk stratification in low-volume nodal disease. Subgroup analyses further showed that the adverse effect of N1b was concentrated mainly in patients with positive ETE, tumor diameter >2 cm, and older age. In contrast, among patients without ETE or with tumors ≤2 cm, the difference in CSD between N1a and N1b disease did not reach statistical significance. This pattern suggests that N1b may function less as a uniformly adverse label and more as a risk marker whose impact becomes amplified in the presence of additional aggressive features.^24^^,^^25^

Our study also underscores the importance of competing-risk analysis in PTC. Because the prognosis is generally favorable, particularly in older patients, non-cancer death has a meaningful impact on long-term outcome assessment. If conventional survival methods alone are used, non-cancer deaths are treated as censored events and cancer-related risk may be overestimated.^26^ In our analysis, N1b disease was associated with higher cumulative incidences of both CSD and OCD, but the more pronounced and consistent difference remained concentrated in CSD. This suggests that the effect of N1b is not merely a marker of greater overall vulnerability, but is closely linked to thyroid cancer–related mortality.^27^

We also observed an association between RAI and lower CSD. Although directionally consistent with clinical practice, this finding should be interpreted cautiously. SEER does not provide information on RAI dose, indication, timing, postoperative thyroglobulin, or molecular markers, and residual indication bias may persist. Therefore, the association between RAI and lower CSD should be viewed as exploratory rather than causal.^28^^,^^29^ By contrast, the more robust finding remains the independent adverse significance of N1b even in the setting of low nodal burden.

From a clinical perspective, patients with N1b disease and ≤5 metastatic lymph nodes should not be regarded as uniformly low risk simply because the nodal burden is limited. In particular, when N1b coexists with extrathyroidal extension, tumor diameter >2 cm, or older age, its adverse effect becomes more apparent. These findings provide population-based outcome evidence supporting the continued consideration of lateral neck involvement during postoperative risk reassessment. More broadly, postoperative risk assessment in low-volume nodal disease should not rely solely on metastatic lymph node number but should integrate nodal location, tumor aggressiveness, and patient age into a more refined composite stratification.^30^

Several limitations should be acknowledged. First, as a retrospective observational SEER-based study, residual confounding cannot be eliminated. Although we adjusted for confounders using PSM and multivariable Fine–Gray modeling, SEER lacks information on metastatic lymph node size, extranodal extension, postoperative thyroglobulin, structural recurrence, molecular markers such as BRAF and TERT, and detailed TSH management.^31^^,^^32^ Therefore, our study cannot directly validate specific ATA 2025 criteria.^33^ Second, several variables, particularly ETE and multifocality, had substantial missingness. Although unknown values were retained as separate categories to reduce selection bias, this approach cannot replace more sophisticated missing-data modeling. Third, we evaluated mortality rather than structural recurrence, whereas ATA risk stratification was originally developed primarily for recurrence risk. Accordingly, our results should be interpreted as complementary evidence for long-term adverse outcome stratification rather than as a direct substitute for recurrence-based guideline frameworks. Future validation using institutional or multi-institutional clinically annotated cohorts with structural recurrence, postoperative thyroglobulin, imaging surveillance, treatment details, and molecular data is warranted to determine whether the mortality-based signal observed here translates into recurrence-based risk stratification. Finally, SEER-based N1a/N1b classification relies on registry coding and cannot fully distinguish all details of clinically apparent vs pathologically identified nodal disease.^34^

## Conclusion

Among patients with PTC and ≤5 metastatic lymph nodes, N1b stage remained independently associated with worse long-term outcomes, and this adverse effect was mainly concentrated in clinically more aggressive or higher-risk subgroups, particularly those with positive ETE, tumor diameter >2 cm, and older age. These findings suggest that in low-volume nodal metastasis, risk assessment should not rely solely on metastatic lymph node number but should place greater emphasis on the anatomic location of N1b disease and its interaction with tumor aggressiveness. Our results may provide complementary evidence for postoperative risk assessment and follow-up stratification in patients with low-volume N1b disease.

## Supplementary Material

oyag186_Supplementary_Data

## Data Availability

The data analyzed in this study are available from the SEER database of the National Cancer Institute. Access to SEER data is available upon registration and completion of a data-use agreement. Additional analytic materials are available from the corresponding author on reasonable request.
