# Supplementary material for: ATA 2015-2025 risk stratification transition: prognostic significance of N1b disease in papillary thyroid carcinoma with limited nodal burden
Source: Oncologist. 2026 May 12;31(6):oyag186. doi: 10.1093/oncolo/oyag186 (PMC13215381; doi:10.1093/oncolo/oyag186)
Supplement: oyag186_Supplementary_Data [file oyag186_supplementary_data.doc]

**
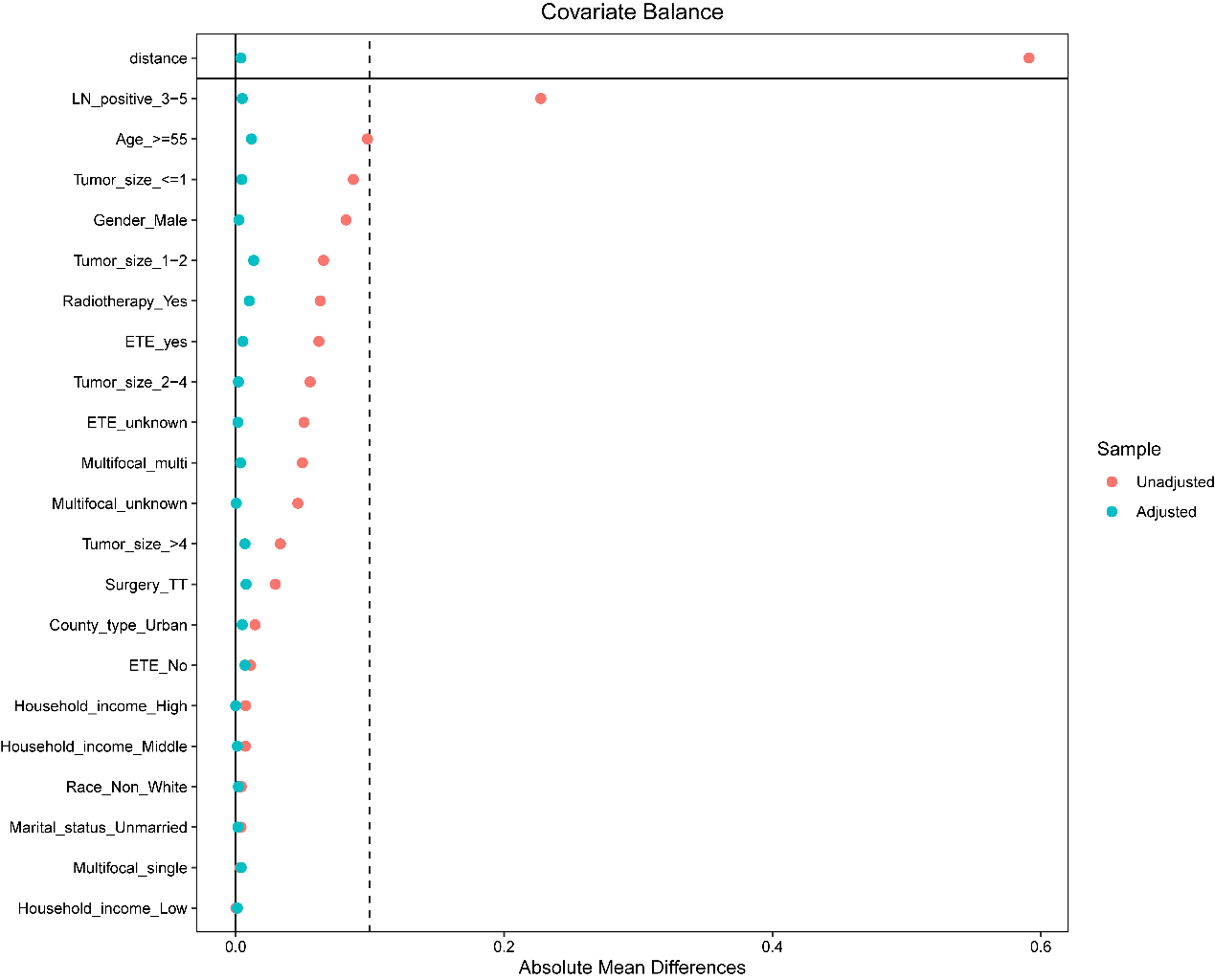
**

**Figure S1. Love plot of covariate balance before and after propensity score matching in patients with PTC and ≤5 metastatic lymph nodes.**

Absolute standardized mean differences are shown for baseline demographic, tumor-related, treatment-related, and socioeconomic variables in the unmatched and matched cohorts. The dashed vertical line denotes an absolute standardized mean difference of 0.10, prespecified as the threshold for acceptable covariate balance.

**Abbreviations:** PTC, papillary thyroid carcinoma; PSM, propensity score matching; ETE, extrathyroidal extension; TT, total thyroidectomy.


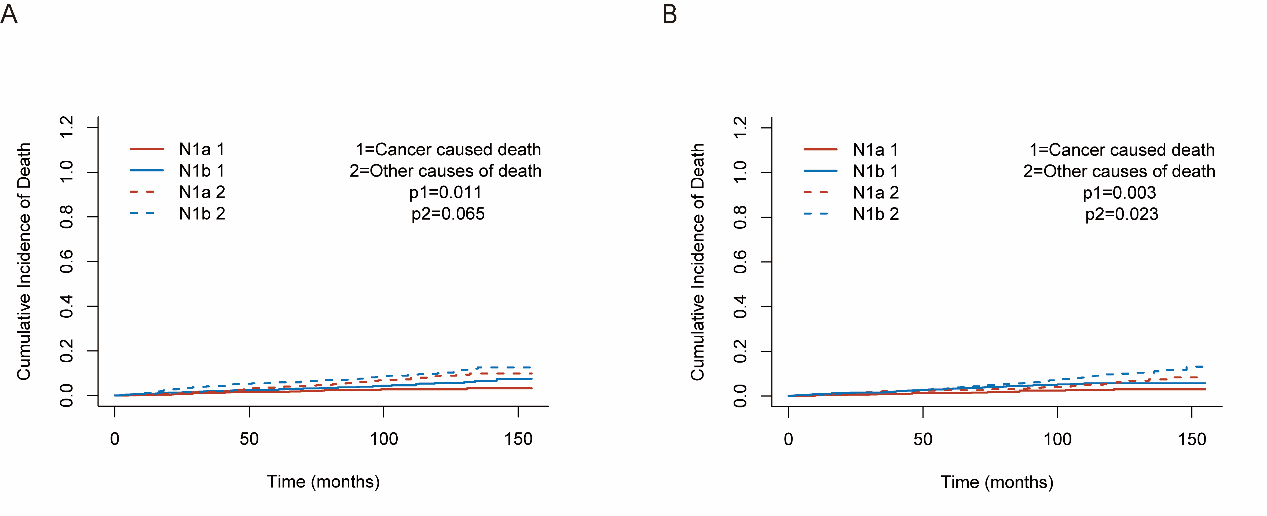


**Figure S2. Cumulative incidence of CSD and OCD by nodal stage stratified by the number of metastatic lymph nodes in the matched cohort of patients with PTC and ≤5 metastatic lymph nodes.**

**(A)** Patients with 1–2 metastatic lymph nodes.
**(B)** Patients with 3–5 metastatic lymph nodes.

Cumulative incidence functions were estimated within a competing-risk framework, and differences between the N1a and N1b groups were compared using Gray’s test.

**Abbreviations:** PTC, papillary thyroid carcinoma; CSD, cancer-specific death; OCD, other-cause death.
